# The cotton laccase gene *GhLAC15 *enhances Verticillium wilt resistance via an increase in defence‐induced lignification and lignin components in the cell walls of plants

**DOI:** 10.1111/mpp.12755

**Published:** 2018-11-15

**Authors:** Yan Zhang, Lizhu Wu, Xingfen Wang, Bin Chen, Jing Zhao, Jing Cui, Zhikun Li, Jun Yang, Liqiang Wu, Jinhua Wu, Guiyin Zhang, Zhiying Ma

**Affiliations:** ^1^ North China Key Laboratory for Germplasm Resources of Education Ministry Hebei Agricultural University Baoding 071001 China

**Keywords:** cell wall composition, defence‐induced lignification, *GhLAC15*, *Gossypium hirsutum*, Verticillium wilt resistance

## Abstract

*Verticillium dahliae* is a phytopathogenic fungal pathogen that causes vascular wilt diseases responsible for considerable decreases in cotton yields. The lignification of cell wall appositions is a conserved basal defence mechanism in the plant innate immune response. However, the function of laccase in defence‐induced lignification has not been described. Screening of an SSH library of a resistant cotton cultivar, Jimian20, inoculated with *V. dahliae *revealed a laccase gene that was strongly induced by the pathogen*.* This gene was phylogenetically related to *AtLAC15* and contained domains conserved by laccases; therefore, we named it *GhLAC15*. Quantitative reverse transcription‐polymerase chain reaction indicated that *GhLAC15* maintained higher expression levels in tolerant than in susceptible cultivars. Overexpression of *GhLAC15 *enhanced cell wall lignification, resulting in increased total lignin, G monolignol and G/S ratio, which significantly improved the Verticillium wilt resistance of transgenic Arabidopsis. In addition, the levels of arabinose and xylose were higher in transgenic plants than in wild‐type plants, which resulted in transgenic Arabidopsis plants being less easily hydrolysed. Furthermore, suppression of the transcriptional level of *GhLAC15* resulted in an increase in susceptibility in cotton. The content of monolignol and the G/S ratio were lower in silenced cotton plants, which led to resistant cotton cv. Jimian20 becoming susceptible. These results demonstrate that *GhLAC15* enhances Verticillium wilt resistance via an increase in defence‐induced lignification and arabinose and xylose accumulation in the cell wall of *Gossypium hirsutum. *This study broadens our knowledge of defence‐induced lignification and cell wall modifications as defence mechanisms against *V. dahliae*.

## Introduction

Allotetraploid upland cotton (*Gossypium hirsutum* L.) is an important cash crop and supplies renewable textile fibres as well as oilseed worldwide. Verticillium wilt, caused by the soil‐borne fungus *Verticillium dahliae* Kleb., stands out amongst the most serious biotic constraints affecting cotton (Bolek *et al*., 2005; Cai *et al*., 2009). The acreage of Verticillium wilt‐infected cotton fields in China is approximately 2.5 million hectares annually, which is equivalent to approximately 50% of the cotton planting area in the country and direct economic losses of approximately US$250–310 million (Li CH *et al*., 2015). Verticillium wilt has not been effectively controlled because of the long‐term existence of microsclerotia and its resting structures in host xylem vessels (Bell, 1992). No fungicide is available to cure commercial upland cotton once infected (Wang *et al*., 2016; Zhang *et al*., 2017).

A promising and environmentally friendly strategy to reduce the above losses is to enhance the immune system of plants via genetic engineering, and this is based on insights into the molecular mechanisms of interactions between plant and pathogen (Lacombe *et al*., 2010). Plants have evolved multiple systems to recognize pathogen attacks. The first layer involves the cell surface perception of conserved microbial components (i.e. pathogen‐/microbe‐associated molecular patterns) and pathogen‐generated plant signal molecules (damage‐associated molecular patterns) by pattern recognition receptors, and the subsequent activation of pattern‐triggered immunity or basal immunity (Boller and Felix, 2009; Zipfel, 2014). The second layer involves the perception of race‐specific pathogen effectors on the cell surface and in the cytosol by resistance (R) proteins, and the subsequent activation of effector‐triggered immunity or R‐mediated immunity (Jones and Dangl, 2006). The downstream responses triggered by these two innate immune strategies are partially overlapping, including the accumulation of secondary metabolites, phytohormones and reactive oxygen species, which activate the corresponding defence systems (De Vleesschauwer *et al*., 2014; Feng and Shan, 2014; Huang *et al*., 2011a,b; La Camera *et al*., 2004; Pieterse *et al*., 2009; Wu *et al*., 2014). More importantly, the pathogen elicitation of basal or R‐mediated immunity has been shown to activate the biosynthesis and deposition of lignin in cell wall appositions (CWAs) (Adams‐Phillips *et al*., 2010; Chezem *et al*., 2017; Kishi‐Kaboshi *et al*., 2010; Lee *et al*., 2001; Robertsen, 1986).

To protect against pathogen infection, plants have evolved multiple sophisticated defence mechanisms (Zhang *et al*., 2017). To overcome the barrier of the plant cell wall, phytopathogenic fungi secrete various cell wall‐degrading enzymes (CWDEs), such as cellulases, pectinase, hemicellulases, cutinase and protease. Most of these enzymes not only degrade cell wall components to obtain carbon sources for pathogen growth, but can also trigger multiple plant defence responses (Tayi *et al*., 2016). As lignin is a very difficult biopolymer to degrade because of the nature and heterogeneity of its linkages (Vanholme *et al*., 2010), it is regarded as a component of the defence response in plants (Miedes *et al*., 2014). For example, lignin has been reported to function as a defensive physical/chemical barrier to limit pathogen colonization or restrict pathogen growth (Bonello and Blodgett, 2003; Zhang *et al*., 2017). Thus, defence‐induced lignification is a conserved basal defence mechanism in the plant immune response against (hemi)biotrophic pathogens in a wide range of plant species (Baayen *et al*., 1996; Bhuiyan *et al*., 2009; Lange *et al*., 1995; Menden *et al*., 2007; Nicholson and Hammerschmidt, 1992; Siegrist *et al*., 1994; Smit and Dubery, 1997; Vance, 1980), and has been used as a biochemical marker of an activated immune response (Adams‐Phillips *et al*., 2010; Kishi‐Kaboshi *et al*., 2010). Previous studies manipulating lignin content and composition have primarily focused on the regulation of the monolignol pathway controlled by 11 enzymatic steps (Vanholme *et al*., 2013). However, genetic alterations in the monolignol pathway impact both plant growth and defence/resistance to hemibiotrophic pathogens (Bhuiyan *et al*., 2009), and cannot distinguish between cell wall lignification and defence‐induced lignification. For example, in Arabidopsis and tobacco (*Nicotiana tabacum*), loss or down‐regulation of phenylalanine ammonia lyase (PAL) leads to decreased basal immunity to the hemibiotrophic bacterial pathogen *Pseudomonas syringae* (Huang *et al*., 2010) and to the biotrophic viral pathogen *Tobacco mosaic virus* (Elkind *et al*., 1990; Pallas *et al*., 1996), respectively. In addition, PAL is also involved in the biosynthesis of the defence signal molecule salicylic acid (SA), which mediates local and systemic resistance to many (hemi)biotrophic pathogens (Sticher *et al*., 1997). Thus, it remains unclear whether the reduced resistance in PAL‐deficient plants is the result of significant reductions in lignin, SA or both. Recently, Hu *et al*. (2018) have reported that the overexpression of cotton *GhLAC1* increases lignification and mediates jasmonic acid (JA) biosynthesis and the balance of the JA–SA defence response, resulting in a modulation of broad‐spectrum biotic stress tolerance. However, whether the improved resistance of transgenic plants is the result of significant increases in lignin, SA, JA or all three still remains unclear.

Lignin from angiosperms usually contains G and S units with low to trace amounts of H units (Davin and Lewis, 1992). S‐rich lignin is less condensed and more degradable (Skyba *et al*., 2013). G‐rich lignin is more cross‐linked and resistant to depolymerization than S‐rich lignin. The ratio of G to S units in lignin indicates the degree and nature of its polymeric cross‐linking (Chezem *et al*., 2017). G‐lignin should be a better defensive barrier against pathogen attack. For example, *AtMYB15* mediates the defence‐induced synthesis of G‐lignin, which contributes to basal immunity in Arabidopsis (Chezem *et al*., 2017). However, several reports have shown inconsistent conclusions on the role of G‐rich lignin in basal immunity. For example, enhancing the G‐lignin level by genetic manipulation of the monolignol pathway in Arabidopsis led to a reduction, not an increase, in R‐mediated immunity to a hemibiotrophic bacterial pathogen (Goujon *et al*., 2003; Quentin *et al*., 2009). In wheat, a higher S‐lignin content is regarded as a cell wall biochemical trait related to Fusarium resistance (Lionetti *et al*., 2015). No genetic studies have been reported providing insights into the role of G‐ or S‐lignin in CWA‐associated defence against *V. dahliae *attack.

By scanning the transcriptomic data in the SSH library that reflects differential expression from Jimian20 root tissue inoculated with *V. dahliae* (Wang *et al*., 2008), we identified one expressed sequence tag (EST) that was homologous to the sequence of *GaLAC* (AY423714). The expression level of this EST, *GhLAC*, was strongly and significantly greater in *V. dahliae*‐infected plants than in plants receiving mock treatment, and the transcription level of *GhLAC* in resistant Jimian20 was significantly higher than that in susceptible Han208 at each time point (Wu *et al*., 2014a). Therefore, we carried out further related experiments to obtain insights into its function in this study. We found that *GhLAC *displayed a differential expression level between tolerant and susceptible cotton varieties, was involved in defence‐induced lignification and modulated Verticillium wilt resistance through an alteration in the G‐rich lignin level and cell wall composition.

## Results

### The GhLAC15 protein is phylogenetically related to the AtLAC15 laccase

Using the EST sequence combined with a previously established full‐length cDNA library platform (Zhang *et al*., 2010), we obtained the full‐length cDNA of *GhLAC* (GI: EU642559.1) from Jimian20, which contained 1701 bp in length. The genome sequences of *GhLAC *contained 2401 bp in length. To identify the homology between GhLAC and laccases from Arabidopsis, we blasted the amino acid sequence of GhLAC against those of 17 Arabidopsis laccase proteins. This phylogenetic analysis showed that the laccases could be divided into two groups. Some sequences (GhLAC, AtLAC14, AtLAC15 and PtLAC) were placed together in the same clade, whereas the others were placed within another clade. The GhLAC protein was especially closely related to the lignin‐specific AtLAC15 laccase; thus, we specifically renamed it *GhLAC15 *(Fig. S1, see Supporting Information). A search for amino acid sequence motifs of laccases showed that the GhLAC15 protein included one transmembrane domain, 13 *N*‐glycosylation sites, three copper oxidase‐like domains, one possible calmodulin‐binding region, one cell attachment sequence, one multicopper oxidase signature and other motif models (Fig. S2, see Supporting Information). A more detailed analysis based on the amino acid sequences was performed by multiple alignments. As shown in Fig. S2, despite the low sequence identity among these proteins, the domains of the cell attachment sequence (Arg‐Gly‐Asp, RGD), the multicopper oxidase signature (HCHLERHSSWGM) and three copper oxidase‐like domains were highly conserved. In addition, *GhLAC15 *was predominantly expressed in the roots, with significantly lower expression levels in stem and leaf (Fig. S3, see Supporting Information).

### *GhLAC15 *displays different expression levels in tolerant and susceptible cotton

**Figure 1:**
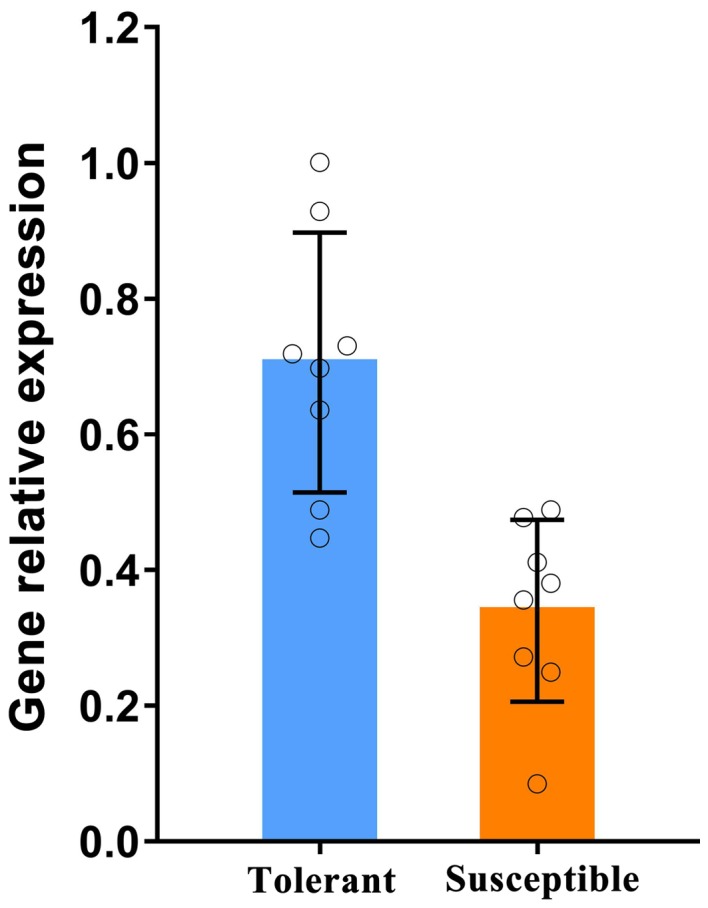
Expression of *GhLAC15* in tolerant and susceptible cotton varieties, detected through quantitative reverse transcription‐polymerase chain reaction (qRT‐PCR). *GhActin* was used as an internal control. Tolerant and susceptible varieties included Tang mian7401, Zhong1421, Su yuan04‐3, Yu mian21, Jin mian20, Su mian22, CC28, Ku che96515 (resistant varieties), Jin zhou tui hua mian, 73‐782, Xu zhou1818, Ren dong67‐86, Xin lu zao28, 73‐184, Nong lin1 and Jun mian1 (susceptible varieties). Data are represented as average values with standard deviation (*n* = 8 varieties with three technical replicates). [Colour figure can be viewed at wileyonlinelibrary.com]

**Figure 2:**
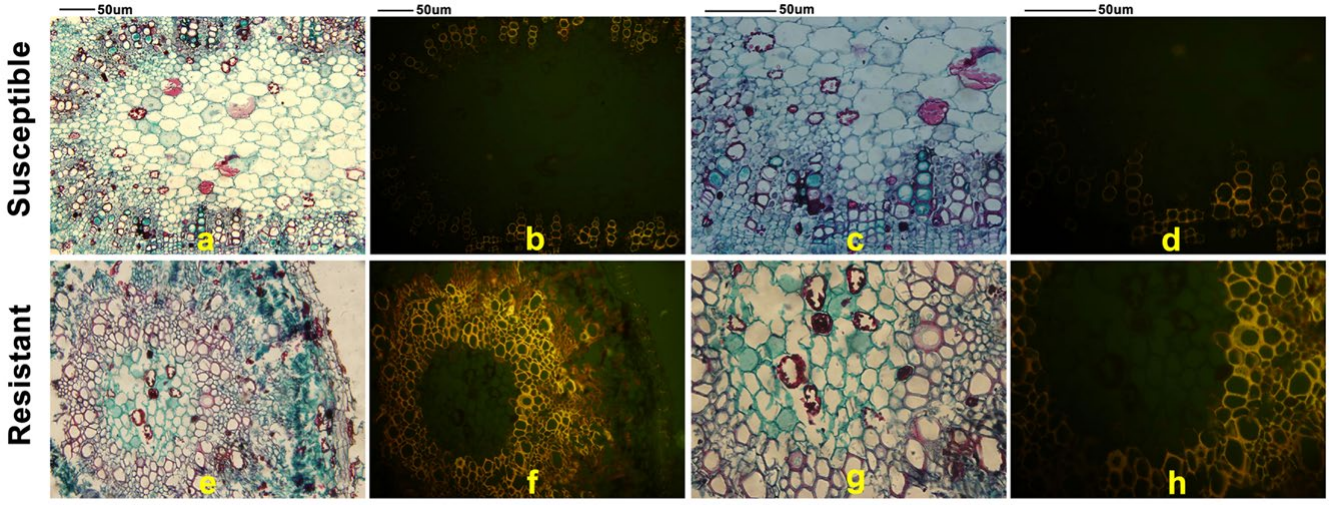
Basic fuchsin staining of cotton stem tissues. Images of susceptible variety cv. Han208 under white light (a, c) and fluorescent light (b, d). Images of resistant variety cv. Jimian20 under white light (e, g) and fluorescent light (f, h). Cross‐sections of stems from the same position of five seedlings were used for this assay. [Colour figure can be viewed at wileyonlinelibrary.com]

To investigate the expression levels of *GhLAC15 *amongst different Verticillium wilt‐resistant cottons, we used two types of varieties, one with a tolerant phenotype and one with a susceptible phenotype Table S2. As shown in Fig. S4 (see Supporting Information), primer efficiencies for *GhLAC15 *and *GhActin *were detected. The results indicated that *GhLAC15 *was more highly expressed in tolerant varieties than in susceptible varieties (Fig. 1). We further detected the abundance of lignin in the resistant variety Jimian20 and susceptible variety Han208, with higher *GhLAC15 *expression in the former than in the latter (Wu *et al*., 2014a), via basic fuchsin staining. As shown in Fig. 2, a greater area of stem tissue was stained with red (an indicator of lignification) in Jimian20 than in Han208, especially in interfascicular regions, and the basic fuchsin fluorescence intensity was also stronger in Jimian20 than in Han208. This difference suggested that the tolerant cotton accumulated more lignin or increased lignification on *V. dahliae* infection.

### GhLAC15 protein is located in the cell wall

**Figure 3:**
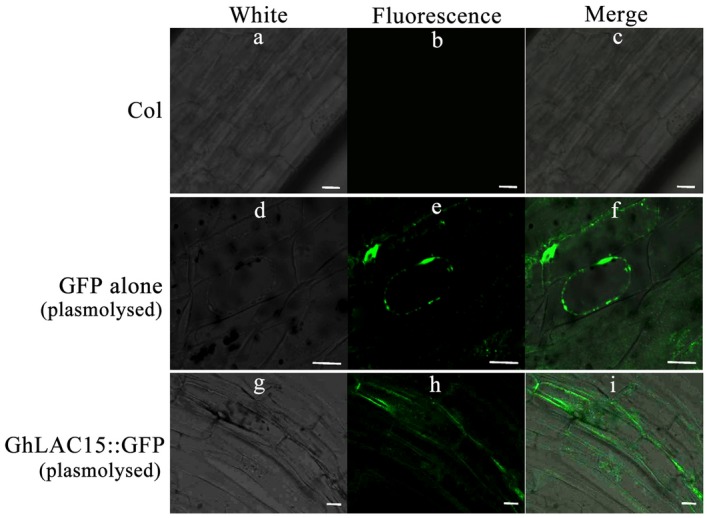
Subcellular localization of the GhLAC15::GFP fusion protein in the root cells of transgenic Arabidopsis. (a–c) Images of wild‐type (d–f) show GFP alone (plasmolysed) and (g–i) show GhLAC15::GFP (plasmolysed). Bar, 50 μm. [Colour figure can be viewed at wileyonlinelibrary.com]

A *GhLAC15* open reading frame (ORF) lacking the stop codon was ligated upstream of an enhanced green fluorescent protein (GFP) gene under the control of the constitutive promoter CaMV 35S. As shown in Fig. 3, the GhLAC15::GFP fusion protein was located in the cell wall of transgenic Arabidopsis and clearly exhibited fluorescence in the cell wall after being plasmolysed with 10% mannitol. As a control, Arabidopsis transformed with the GFP gene alone showed a very strong fluorescence signal, and the signal was uniformly and diffusely distributed in the cytosol and nucleus, as described by Cutler *et al*. (2000); fluorescence was absent in wild‐type (WT) Arabidopsis as expected. These results thus suggested that the GhLAC15 protein was localized to the cell wall.

### *GhLAC15 *enhances the resistance of transgenic Arabidopsis against *V. dahliae*

**Figure 4:**
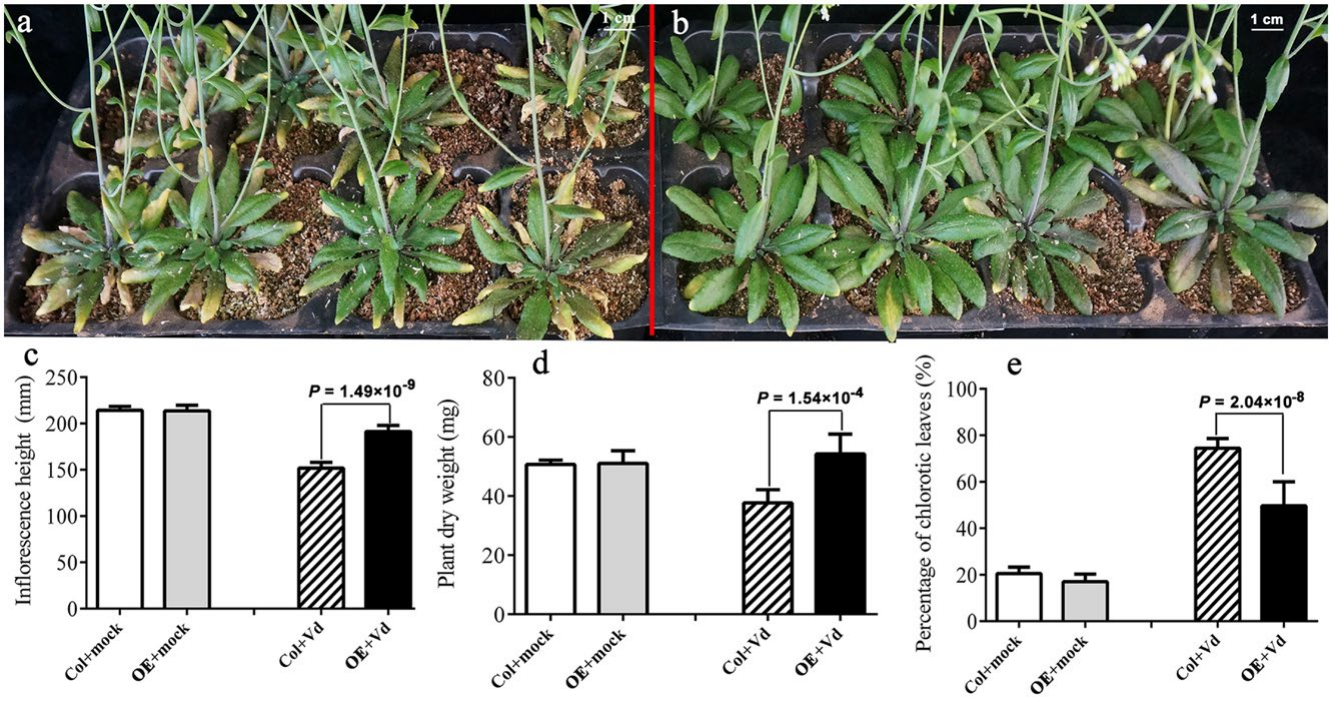
Overexpression of *GhLAC15* in Arabidopsis improved resistance to *Verticillium dahliae*. (a, b) Four‐week‐old plants were inoculated by root dipping with 5 × 10^7^ conidia/mL. Transgenic (b) and wild‐type (a) plants showed marked differences in Verticillium wilt resistance. Transgenic plants were more resistant than wild‐type plants. (c–e) Disease resistance was assessed by measurement of the inflorescence height (c) and plant dry weight (d), as well as the percentage of leaf chlorosis (e). The significance of differences was analysed with the *t*‐test (*P* ＜ 0.01). Data are presented as average values with standard deviation (*n* = 39). Col, wild‐type Arabidopsis; OE, transgenic Arabidopsis. [Colour figure can be viewed at wileyonlinelibrary.com]

**Figure 5:**
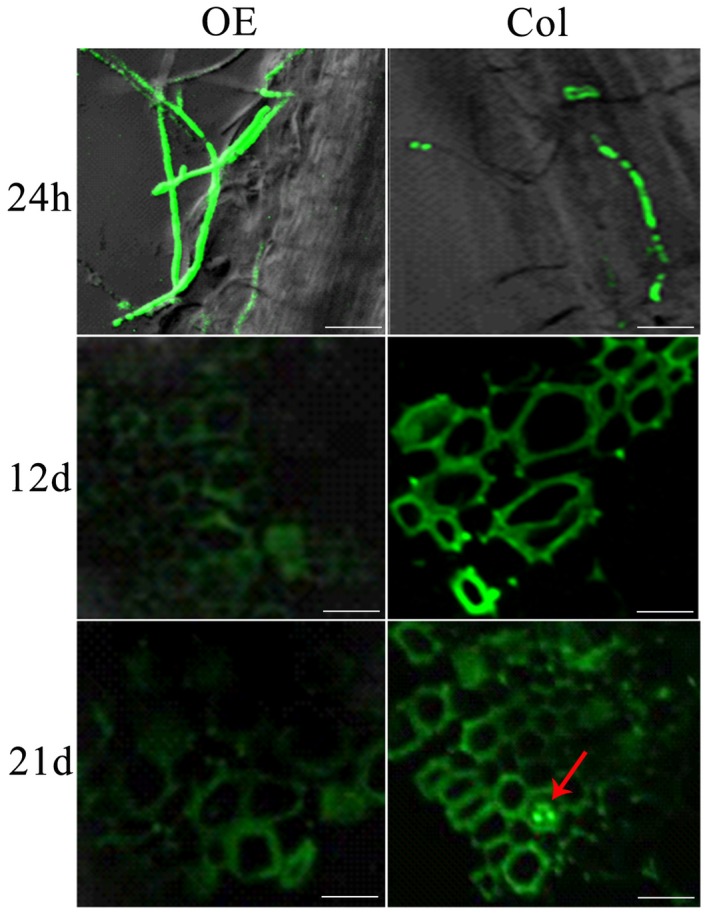
Progression of root colonization by green fluorescent protein (GFP)‐tagged isolate of *Verticillium dahliae* in transgenic Arabidopsis (OE) and wild‐type plants (Col). Representative images of conidiospores were taken at 24 h, 12 days and 21 days. The red arrow points to the accumulation of *V. dahliae* in the vascular bundle. Ten mature rosette leaves of each transgenic line were observed, and three transgenic lines were used for this assay. Bar, 20 µm. [Colour figure can be viewed at wileyonlinelibrary.com]

To validate the function of the *GhLAC15* gene, we first overexpressed *GhLAC15* in Arabidopsis. Of the 16 independent T_3_ transgenic lines, three stable overexpressing lines selected via genome PCR and RT‐PCR analysis were inoculated with *V. dahliae*. Disease resistance was assessed by evaluation of the extent of leaf chlorosis and measurement of inflorescence heights and plant dry weights. Typical symptoms of vascular disease became evident in the infected WT plants, but were much less pronounced in transgenic plants at 20 days post‐inoculation (dpi) (Fig. 4a). Compared with WT, transgenic Arabidopsis exhibited significantly more resistance (Fig. 4b). The average inflorescence height of inoculated WT plants (155.3 cm) was significantly shorter than that of transgenic plants (199.5 cm), and the average dry weight of infected WT plants was lower than that of transgenic plants (Fig. 4c,d). The extent of leaf chlorosis also differed markedly between WT and transgenic plants. By comparison, disease symptoms developed more slowly and were less severe in transgenic plants than in WT (Fig. 4e). In detail, the percentage of chlorotic leaves in transgenic plants was nearly 50%, and only minor microsclerotium formation was observed in the vascular bundles (Fig. 5). However, more than 71% of WT leaves experienced chlorosis, and greater microsclerotium formation occurred in the vascular bundles of WT plants (Figs 4e and 5). Based on these symptoms, we concluded that *GhLAC15 *enhanced the Verticillium wilt resistance of transgenic Arabidopsis.

### The *GhLAC15* expression levels positively correlate with resistance against *V. dahliae *in *G. hirsutum*

**Figure 6:**
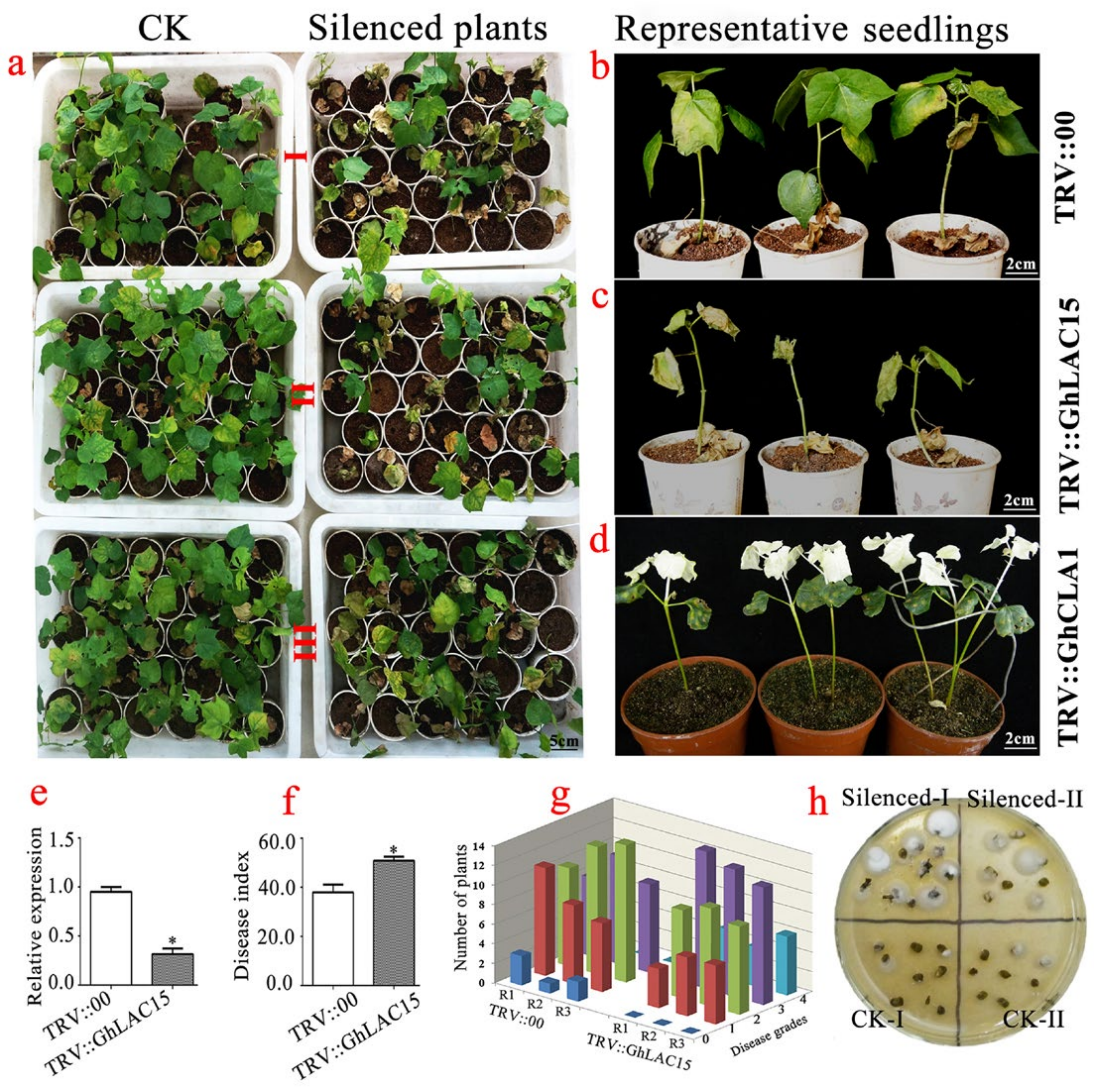
Effects of silencing of *GhLAC15 *on cotton susceptibility to *Verticillium dahliae*. Two weeks after infiltration, seedlings were inoculated with *V. dahliae*. (a) Responses of control (CK) (TRV::00) and silenced (TRV::GhLAC15) plants to the pathogen at 20 days post‐inoculation (dpi). Disease symptoms induced on CK and silenced plants with three experimental repeats. (b, c) Representative seedlings of CK and silenced plants after inoculation with *V. dahliae* at 20 dpi. (d) Seven‐day‐old cotton plants were infiltrated with *Agrobacterium* carrying TRV::GhCLA1. The photographs were taken at 2 weeks after infiltration. (e) Preliminary assay of the efficiency of virus‐induced gene silencing (VIGS) under our experimental conditions. (f, g) The disease index and rate of diseased plants were measured at 20 dpi. Error bars represent the standard deviation of three biological replicates (*n* = 36); asterisks indicate statistically significant differences, as determined by *t*‐test (*P* < 0.05). (h) Fifteen days after *V. dahliae* inoculation, surface‐sterilized hypocotyl sections prepared from CK and silenced plants were placed on agar medium at 7 dpi. The number of stem sections from which fungus grew represented the extent of fungal colonization. [Colour figure can be viewed at wileyonlinelibrary.com]

Virus‐induced gene silencing (VIGS) is a promising approach in plant functional genomics and has been widely used to study gene function in cotton (Gao *et al*., 2011). Therefore, we employed VIGS to investigate the function of *GhLAC15* in defence against *V. dahliae* infection. As shown in Fig. 6d, an albino phenotype appeared on newly developing true leaves and stems of plants infiltrated with agrobacteria carrying *GhCLA1*, indicating that the VIGS system worked efficiently under our experimental conditions. Meanwhile, quantitative reverse transcription‐polymerase chain reaction (qRT‐PCR) analyses showed that the expression of *GhCLA1* in VIGS plants was significantly lower than that in plants with the empty vector (Fig. 6e). When plants were challenged with *V. dahliae*, the down‐regulation of *GhLAC15* expression resulted in reduced resistance to the pathogen (Fig. 6a–c). The disease index and rate of diseased plants were clearly greater in *GhLAC15*‐silenced plants than in non‐silenced plants (Fig. 6f,g). Larger numbers of fungal colonies were found in the roots of *GhLAC15*‐silenced plants, suggesting that the extent of fungal colonization was much more severe in *GhLAC15*‐silenced plants than in control plants (Fig. 6h).

To clearly understand the relationship between changes in *GhLAC15* expression and disease resistance in VIGS plants, the gene expression level and the corresponding disease resistance were investigated in 30 VIGS plants (Fig. S5, see Supporting Information). A reduction in *GhLAC15 *gene expression was positively correlated with aggravated susceptibility to *V. dahliae*. This result suggested that *GhLAC15* played an important role in defence against *V. dahliae* in cotton.

**Figure 7:**
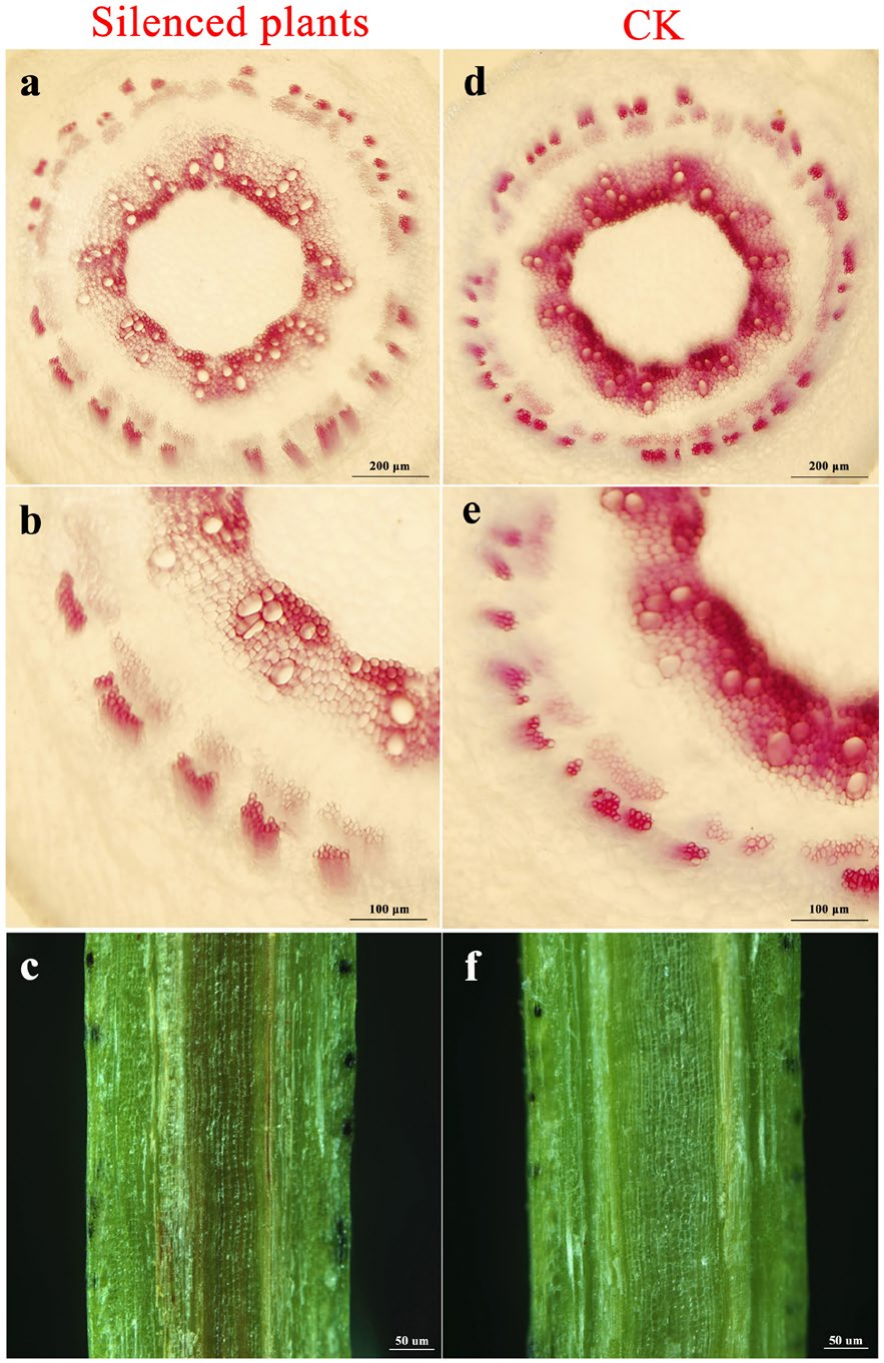
Determination of lignin in virus‐induced gene silencing (VIGS)‐treated cotton after inoculation with *Verticillium dahliae*. Light microscopy image of hypocotyl cross‐sections at 4 days post‐inoculation (dpi) after staining with phloroglucinol‐HCl to detect lignin in silenced plants (a, b) and control (CK) plants (d, e). At 20 dpi, tissue browning was rare in vascular bundles from CK plants (f), but severe in longitudinal sections from silenced plants (c). Cross‐sections of stem from the same position of five seedlings were used for the assays. [Colour figure can be viewed at wileyonlinelibrary.com]

We further investigated whether lignin deposition or monolignol composition was altered after silencing of *GhLAC15*. The histochemical staining results indicated that, at 4 dpi, hypocotyl sections from GhLAC15‐silenced plants exhibited lighter red staining of xylem vessel walls and parenchyma cell walls than control plants (Fig. 7a,b,d,e). In addition, the stem of *GhLAC15*‐silenced plants exhibited darker vascular browning (Fig. 7c,f). Monolignol composition analysis showed that both G‐ and S‐lignin levels, as well as the G/S ratio, decreased significantly after *GhLAC15* was silenced in cotton (Table 1), which suggested that *GhLAC15* functioned to modulate monolignol composition biosynthesis and the G/S ratio, thus regulating cotton Verticillium wilt resistance.

**Table 1:** Monolignol composition in cell walls from control (CK) and silenced cotton plants.

Line	Thioacidolysis yield (μmol/g extract‐free sample)			G/S molar ratio
	G	S	H	
CK plant	135.0 ± 6.9^a^	72.6 ± 3.3^a^	8.8 ± 0.6	1.86 ± 0.09^a^
Silenced plant	87.1 ± 5.4^b^	52.9 ± 4.0^b^	9.3 ± 0.4	1.65 ± 0.02^a^

The data are the mean ± standard error (SE) of three independent biological replications, and different letters indicate significant differences at *P* < 0.05 (Duncan’s multiple range test).

### *GhLAC15 *alters lignification and enzymatic hydrolysis in mature transgenic Arabidopsis stems

To confirm whether *GhLAC15* functions in lignin polymerization, we determined the lignin content by measurement of both the Klason lignin (KL) and acid‐soluble lignin (ASL) concentrations of extract‐free stems. Transgenic plants exhibited higher KL content than the corresponding controls under *V. dahliae* stress or free (Table 2). Compared with mock plants, both WT and transgenic plants decreased in KL content after inoculation with *V. dahliae*. However, ΔKL in transgenic plants was 11.36%, significantly lower than that in WT plants (17.76%) (Table 2). ASL lignin contents were similarly low in all samples (within the range of 1%). The results indicated that *GhLAC15 *enhanced KL, which played a crucial role in disease resistance.

**Table 2:** Lignin content of mature stems of Arabidopsis.

Treatment	Line	Lignin content	
		KL (%)	ASL (%)
Mock	Wild‐type	21.12 ± 0.76^b^	2.53 ± 0.24^c^
	OE plant	24.29 ± 0.07^a^	3.17 ± 0.66^b^
Inoculated	Wild‐type	17.37 ± 1.20^c^	2.94 ± 0.06^b^
	OE plant	21.50 ± 0.85^b^	3.46 ± 0.00^a^
ΔLignin	Wild‐type	－17.76%	＋16.21%
	OE plant	－11.36%	＋9.15%

ASL, acid‐soluble lignin; KL, Klason lignin; OE, overexpressing. The data are the mean ± standard error (SE) of three independent biological replications, and different letters in the same column share a significant difference (*P* < 0.05) (Duncan’s multiple range test). ΔLignin = (Inoculated － Mock)/Mock.

**Figure 8:**
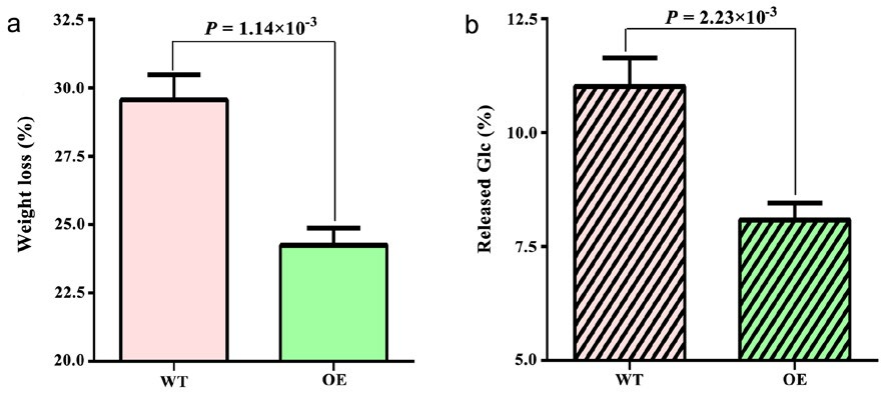
Enzymatic hydrolysis of mature stem from transgenic Arabidopsis. (a) Weight loss after enzymatic hydrolysis in wild‐type (WT) and overexpressing (OE) Arabidopsis. Data are presented as average values with standard deviation (*n* =  3 technical replicates). (b) Glucose (Glc) released after enzymatic hydrolysis of WT and OE Arabidopsis. The significance of differences was analysed with the *t*‐test. Data are presented as average values with standard deviation (*n*  =  3 technical replicates). WT and OE samples displayed significantly different weight loss (*P* = 1.14 × 10^−3^) and amount of released Glc (*P* = 2.23 × 10^−3^). [Colour figure can be viewed at wileyonlinelibrary.com]

Lignin content is known to be a factor in decreasing the cell wall susceptibility to enzymatic hydrolysis. Thus, we subjected the extract‐free samples to treatment with a commercial cellulose preparation to evaluate the saccharification potential of transgenic and WT plants. Consistent with their low KL level, WT plants lost an average of 29.76% of their weight when subjected to commercial cellulose treatment (cellulose and hemicellulase activities), which was much greater than that observed in transgenic plants. By contrast, transgenic plants were less easily hydrolysed than WT plants, with less weight loss (Fig. 8a). The amount of glucose (Glc) released by enzymatic hydrolysis was also significantly lower in transgenic plants (Fig. 8b). Thus, we concluded that the modified cell wall traits could function as a defence barrier to improve the resistance of Arabidopsis to *V. dahliae* infection.

We further determined the impact of overexpression of *GhLAC15 *on lignin structure by thioacidolysis using entire mature inflorescence stems of 6‐week‐old plants (Table 3). The H thioacidolysis monomer will not be discussed further because of its small amounts recovered in all samples. As shown in Table 3, under normal conditions (free of *V. dahliae*), transgenic plants released significantly larger amounts of guaiacyl (G) monomers than WT, and equal amounts of syringyl (S) monomers as WT, indicating the crucial role of *GhLAC15* in G‐lignin biosynthesis. On inoculation, the G monomer content was much higher in transgenic plants than in WT plants, even though there was a marked decrease in both transgenic and WT plants. The S monomer content in transgenic plants remained unchanged, whereas it decreased sharply in WT plants post‐inoculation with *V. dahliae*. Compared with its value in transgenic plants, the G/S ratio obviously decreased in WT after inoculation by *V. dahliae* (Table 3). The results suggested that *GhLAC15* could maintain a relatively higher ratio of G‐units to S‐units in lignin, thus conferring improved Verticillium wilt resistance in transgenic plants (Xu *et al*., 2011).

**Table 3:** Determination of the main G, S and H thioacidolysis monomers released by the lignin of extract‐free mature stems.

Treatment	Line	Thioacidolysis yield (μmol/g extract‐free sample)				G/S molar ratio
		G	S	H	Total	
Mock	Wild‐type	203.1 ± 0.0^b^	74.0 ± 0.0^a^	2.0 ± 0.0	252.2 ± 33.9^b^	3.15 ± 0.05^a^
	OE plant	225.6 ± 0.0^a^	77.2 ± 0.0^a^	2.2 ± 0.0	314.0 ± 30.8^a^	2.71 ± 0.21^a^
Inoculated	Wild‐type	62.0 ± 1.5^d^	35.8 ± 0.2^b^	1.3 ± 0.0	99.1 ± 1.8^c^	1.64 ± 0.20^b^
	OE plant	176.4 ± 16.5^c^	77.9 ± 11.7^a^	1.9 ± 0.6	256.1 ± 28.9^b^	2.28 ± 0.13^a^
ΔLignin	Wild‐type	−69.5%	−51.6%	‐	−60.7%	‐
	OE plant	−21.8%	＋0.9%	‐	−18.4%	‐

The data are the mean ± standard error (SE) of three independent biological replications, and different letters in the same column share a significant difference (*P* < 0.05) (Duncan’s multiple range test). ΔLignin = (Inoculated − Mock)/Mock. OE, overexpressing.

### *GhLAC15 *increases total lignin, arabinose (Ara) and xylose (Xyl) in transgenic Arabidopsis

The total lignin contents of the entire mature inflorescence stems were determined by the acetyl bromide method to further confirm the impact of the overexpression of *GhLAC15* on lignin biosynthesis. The results indicated that the transgenic plants exhibited significantly higher total lignin content than WT whether under *V. dahliae* stress or free (Table 3). Compared with mock plants, total lignin content decreased in both WT and transgenic plants after inoculation by *V. dahliae*. However, ΔLignin in transgenic plants was 18.4%, significantly lower than that (60.7%) in WT plants (Table 3). Plant cell wall composition and features can greatly affect plant mechanical strength and resistance to pathogens and pests (Bonello and Blodgett, 2003; Mottiar *et al*., 2016; Naoumkina *et al*., 2010). Therefore, we analysed the predominant carbohydrate constituents of the cell wall in transgenic Arabidopsis, including Glc, Ara and Xyl. As shown in Table 4, both Ara and Xyl levels were significantly higher in transgenic Arabidopsis than in WT under *V. dahliae* stress. In contrast with WT, the Glc level showed a remarkable decrease in transgenic Arabidopsis. Thus, the overexpression of *GhLAC15* changed the cell wall composition, leading to increases in Ara and Xyl content.

**Table 4:** Detection of polysaccharide components and content in Arabidopsis.

Line	Polysaccharide components of cell wall (%)			
	Ara content	Xyl content	Glc content	Ara + Xyl
Wild‐type	2.63 ± 0.03^b^	8.87 ± 0.49^b^	2.86 ± 0.27^a^	11.62 ± 0.57^b^
OE plant	3.33 ± 0.08^a^	12.43 ± 0.10^a^	1.70 ± 0.08^b^	15.86 ± 0.00^a^

Ara, arabinose; Glc, glucose; Xyl, xylose. OE, overexpressing. The data are the mean ± standard error (SE) of three independent biological replications, and different letters in the same column share a significant difference (*P* < 0.05) (Duncan’s multiple range test).

## Discussion

The plant cell wall, composed of cellulose, hemicelluloses, lignin and pectic polysaccharides with minor structural proteins, is a dynamic structure that often determines the outcome of the interactions between plants and pathogens (Bellincampi *et al*., 2014; Xu *et al*., 2011). Lignin has been reported to be involved in multiple resistances, including cotton Verticillium wilt resistance (Bonello and Blodgett, 2003; Hu *et al*., 2018; Zhang *et al*., 2017). Previous studies have shown that plant laccases (*LAC4*, *LAC11* and *LAC17*) are necessary and non‐redundant with peroxidase for lignin polymerization in Arabidopsis (Berthet *et al*., 2011; Zhao *et al*., 2013), and that they oxidatively polymerize monolignols (*p*‐coumaryl, coniferyl and sinapyl alcohols) into *p*‐hydroxyphenyl (H), guaiacyl (G) and syringyl (S) lignin units (Wang *et al*., 2015). In this study, we isolated a lignin laccase, GhLAC15, which affected Verticillium wilt resistance by increasing total lignin and KL levels and by modifying lignin composition and structure, as well as associated cell wall traits. These findings provide unprecedented insights into the defence mechanism against *V. dahliae* of cotton laccase genes.

The cell wall is a barrier that pathogens need to breach to colonize plant tissue. During infection, pathogens produce CWDEs, such as pectinases, xylanases and cellulases, to degrade cell wall polysaccharides to penetrate and colonize the host tissues (Wanyoike *et al*., 2002; Yang *et al*., 2012). The role of cell wall components in plant resistance has been reported in different species (Barros‐Riosa *et al*., 2011; Lionetti *et al*., 2015). New lines of evidence have suggested that the content and composition of cell wall polymers affect the susceptibility of cell walls to CWDEs and play important roles in the outcome of host–pathogen interactions (Blümke *et al*., 2014; Cantu *et al*., 2008; Pogorelko *et al*., 2013). Notably, the extent of cell wall degradation is often associated with the severity of diseases (King *et al*., 2011). Lignin is an important structural component involved in defence against invasive pathogens, making the cell wall more resistant to CWDEs and preventing the diffusion of pathogen‐produced toxins (Sattler and Funnell‐Harris, 2013; Zhang *et al*., 2017). In this study, we found that the overexpression of *GhLAC15* increased significantly the lignin content of Arabidopsis, leading to improved Verticillium wilt resistance. Compared with mock plants, both transgenic and WT plants displayed decreased lignin content under *V. dahliae* stress. This may be a result of the degradation of plant cell walls by CWDEs produced by *V. dahliae*. However, the change in lignin amount in transgenic plants was much lower than that in WT plants. In other words, the cell wall of transgenic plants was more resistant to CWDEs produced by *V. dahliae *and probably maintained cell wall‐related integrity or exhibited higher plant mechanical strength.

Lignin is a factor in decreasing the cell wall susceptibility to enzymatic hydrolysis (Berthet *et al*., 2011). In this study, *GhLAC15* transgenic plant stems were less easily hydrolysed. In a previous study, *lac4‐2 *and *lac17 *mutants showed low KL levels and much greater weight loss than WT plants when treated by commercial cellulose, and about one‐half of this loss was accounted for by Glc (Berthet *et al*., 2011). It has been reported that, during host and pathogen interactions, pathogens can degrade the host cell wall by self‐produced CWDEs, resulting in higher Glc levels (Yang *et al*., 2018). When we analysed the predominant carbohydrate constituents of cell walls in transgenic Arabidopsis, the Glc level was significantly lower in transgenic Arabidopsis than in WT; therefore, we concluded that cotton *GhLAC15 *altered the susceptibility of the Arabidopsis cell wall to enzymatic hydrolysis, which might be very important in defence against pathogen infection.

In this study, both Ara and Xyl levels were significantly higher in transgenic Arabidopsis than in WT under *V. dahliae* stress. The Ara level is a positive factor in rice lodging resistance and lignin level (Li F *et al*., 2015), and high concentrations of Xyl play an important role in defence against corn borers and are regarded as a defence mechanism of maize (Barros‐Riosa *et al*., 2011). Furthermore, the higher values for Ara and Xyl represent a high content of arabinose‐substituted xylan (arabinoxylan) (Barros‐Riosa *et al*., 2011). High concentrations of arabinoxylan in papillae effectively restrict pathogen penetration in barley (Chowdhury *et al*., 2014). Thus, we deduced that the increases in Ara and Xyl in Arabidopsis overexpressing *GhLAC15 *contributed to Verticillium wilt resistance.

## Experimental Procedures

### Growth of plant material and pathogen cultures

The *G. hirsutum* L. variety cv. Jimian20, which displays resistance to Verticillium wilt, was used in this study. Seeds of Jimian20 were surface disinfected in 0.5% sodium hypochlorite (NaOCl) for 5 min and then washed five times with sterile water. Any seed presenting internal fungal contamination was discarded. Seedlings of similar size were selected and cultivated in dishes containing sterile vermiculite under the following growth chamber conditions: 16‐h photoperiod, 30 ± 2 °C/25 ± 2 °C (day/night) and 75% relative humidity. Hoagland’s nutrient solution was added to the dishes every 4 days.

The highly aggressive defoliating *V. dahliae* strains Vd991 and Vd‐gfp77 were cultured on potato dextrose agar (PDA) for 7 days at 25 °C. The fungus was then inoculated into Czapek medium and cultured on a shaker at 120–140 *g* at 25 °C for another 3–4 days until the concentration of spores reached approximately 10^8^–10^9^ spores/mL. The suspension was adjusted to an approximate density of 10^7^ conidia/mL with deionized water prior to use. Samples of seedlings inoculated with *V. dahliae* were used for RNA extraction, whereas seedlings inoculated with the gfp77 strain were used for observation of the progress of infection.

### Gene cloning and bioinformatic analysis

Total RNA was extracted from cotton plants using an EASYspin Plus Plant RNA Kit (Aidlab Biotech) according to the manufacturer’s instructions. The purified RNA was used as a template to prepare cDNA with the PrimeScript™ II 1st Strand cDNA Synthesis Kit (TaKaRa). A full‐length *GhLAC15* coding sequence with *Kpn*I and *Xba*I linkers was cloned using the primers LacF1 and LacR1 (Table S1, see Supporting Information).

### Gene expression analysis

To determine the expression level of *GhLAC15 *in different cottons or tissues, the target samples were harvested, quickly frozen in liquid nitrogen, ground into a fine powder and stored at –80 °C until use. Total RNA isolation, cDNA synthesis and qRT‐PCR were performed as described previously (Zhang *et al*., 2017). The cotton GhLAC15‐specific primers LacF2/LacR2 (Table S1) were designed and included the 5′‐untranslated region (UTR) to discriminate the target *GhLAC15* gene from other members of the gene family. Real‐time PCR was performed in triplicate with SYBR Premix Dimer Eraser (Perfect Real Time) (Dlian, China, TaKaRa). *GhActin *served as the reference gene to normalize the total amount of cDNA in each reaction. For each sample, cDNA was serially diluted in sterile water (1, 1 : 2, 1 : 4, 1 : 8, 1 : 16, 1 : 32, 1 : 64 and 1 : 128) to investigate the amplification efficiencies of the primers. Relative fold differences in mRNA abundance were defined by the mathematical model described by Livak and Schmittgen (2001). All experiments were performed with three technical replicates. The primers used for gene expression analysis are listed in Table S1.

### Generation of transgenic Arabidopsis and disease assays

The full‐length ORF of *GhLAC15* was amplified using forward (LacF3) and reverse (LacR3) primers (Table S1) that incorporated *Kpn*I and *Xba*I cleavage sites, respectively. To prepare an overexpression (OE) construct, we inserted the *GhLAC15 *coding region of Jimian20 into the plant binary vector pCAMBIA1301S under the control of the CaMV 35S promoter. This vector was used to transform *Agrobacterium tumefaciens* strain GV3101 using a freeze–thaw method. Positive colonies were confirmed by PCR together with sequencing, and used for the genetic transformation of the Arabidopsis plants using the floral dip method (Clough and Bent, 1998). The identification of transformed seeds was based on hygromycin B screening and PCR detection with the primers LacF3/LacR3. Homozygous T_3_ seeds of transgenic lines were used for phenotypic analyses.

### Fluorescence microscopy

Transgenic Arabidopsis seeds with the GhLAC15::GFP fusion were sterilized and germinated on Murashige and Skoog (MS) medium on a clean bench as described by Clough and Bent (1998). The roots of 7‐day‐old transgenic and WT Arabidopsis were plasmolysed with 10% mannitol (Komis *et al*., 2008). Specimen sections were observed using a fluorescence microscope (DM2500; Leica, Wetzlar, Germany).

### VIGS in cotton and pathogen inoculation

*Tobacco rattle virus* (TRV)‐based VIGS was performed in cotton as described previously (Gao *et al*., 2011). The pTRV1, pTRV2 and pTRV2 derivatives harbouring specific regions of *GhLAC15 *were transformed into *A. tumefaciens* strain GV3101 by electroporation. The primers (LacF4/LacF4) used for *GbLAC15 *fragment amplification are listed in Table S1. Seven‐day‐old seedlings were transformed with a mixture (1 : 1, v/v) of *Agrobacterium* cultures harbouring pTRV1 with pTRV2 or its derivative plasmids. After completion of agro‐inoculation, the seedlings were washed with deionized water to remove excess agrobacterial inoculum and grown at 25 °C under a 16‐h/8‐h light/dark cycle in a controlled environmental chamber. After 2 weeks of cultivation, the plants were inoculated with *V. dahliae*. The experiments were performed with at least 36 seedlings per treatment and were repeated three times. The rate of diseased plants and the disease index were calculated as described previously (Zhang *et al*., 2016).

### Lignin histochemical staining

Hypocotyl samples were taken at 3 dpi from 10 inoculated and 10 mock‐treated plants of each species. For histochemical analysis, 2‐cm‐long segments were excised and preserved in a mixture of acetic acid–formalin–ethanol (5 : 5 : 90, v/v/v). Tissues were transverse cross‐sectioned on a vibration microtome (VT 1000M; Leica). Lignin histochemistry was examined using Wiesner reagent (Pomar *et al*., 2004) or basic fuchsin staining. The cross‐sections were incubated for 10 min in a phloroglucinol solution (2 in 95% ethanol) or 95% ethanol (staining control), and then treated with 18% HCl for 5 min, and directly observed under bright‐field conditions with a fluorescence microscope (DM2500; Leica). Basic fuchsin staining of stem sections was performed according to the protocol of Kapp *et al*. (2015).

### Determination of ASL and KL contents

Lignin chemical analysis was performed based on the above‐ground parts of Arabidopsis plants. Arabidopsis plants were collected and vacuum freeze‐dried for 48 h. Samples were evenly ground to pass through a 0.5‐mm sieve before an exhaustive solvent extraction, first with toluene–ethanol (2 : 1, v/v), then with ethanol and finally with water, as described previously (Berthet *et al*., 2011; Lapierre *et al*., 1995). The amounts of KL and ASL (spectrophotometric method) were determined by the Klason method with modifications (Dence, 1992). For each sample, 3 mL of 72% (w/w) sulfuric acid were added to 300 mg of the sample in a pressure tube. The samples were incubated in a water bath at 30 °C for 1.5 h with intermittent mixing using a glass rod. On completion of hydrolysis, the acid solution was diluted to a 4% concentration by the addition of 84 mL of water. The samples were mixed well by inverting the tube several times, and the Teflon caps were screwed on securely. The pressure tubes were then placed in an autoclave for 1 h using the ‘liquids’ setting (121 °C). The autoclaved hydrolysis solution was vacuum filtered. All remaining acid‐insoluble lignin was KL. The content was calculated as the weight percentage of the extract‐free wood and reported as the average of at least three independent determinations on the same sample. The filtrate was used to quantify ASL with a spectrophotometer. The maximal absorbance of the diluted solution was measured at 320 nm as described by Sluiter *et al*. (2013).

### Determination of lignin content and composition

Mature stems were harvested for lignin analysis according to published procedures (Zhao *et al*., 2010). Lignin‐derived compounds were identified by analysis of their trimethylsilyl derivatives using gas chromatography/mass spectrometry. Lignin analyses were performed with three independent transformed lines and three biological replicates for lignin quantification and lignin composition. Statistical analysis was performed using the *t*‐test (*P* < 0.01 or *P* < 0.05).

## Conflicts of Interest

The authors have declared that no competing interests exist.

## Supporting information


**Fig. S1** Phylogenetic analysis of laccase proteins. Phylogenetic relationship of GhLAC15, AtLAC proteins and two other plant laccases. The phylogenetic tree was constructed using DNAMAN6.0 Multiple Sequence Alignment programs. GhLAC15 is marked in red. The two letters preceding the protein name describe the organism from which the sequence was derived: At, *Arabidopsis thaliana*; Pt, *Populus trichocarpa*. LAC, laccase. AtLAC1 (NM_101674), AtLAC2 (NM_128470), AtLAC3 (NM_128574), AtLAC4 (NM_129364), AtLAC5 (NM_129597), AtLAC6 (NM_130222), AtLAC7 (NM_111756), AtLAC8 (NM_120181), AtLAC9 (NM_120182), AtLAC10 (NM_120197), AtLAC11 (NM_120404), AtLAC12 (NM_120621), AtLAC13 (NM_120795), AtLAC14 (NM_120972), AtLAC15 (NM_124184), AtLAC16 (NM_125281), AtLAC17 (NM_125395), PtLac (XM_002325536). The bar indicates the relative branch length. GhLAC15 and GhLAC17 laccases from Arabidopsis were used to build the tree. The branch length is proportional to the number of substitutions per site and represents evolutionary distance, as indicated by the scale bar.

**Fig. S2** Multiple alignment of the deduced amino acid sequences of GhLAC15 and other plant laccases. The amino acid sequences of GhLAC15, GaLAC, PtLAC and *Arabidopsis thaliana* (AtLAC4 and AtLAC17) were aligned with ClustalW software. The consensus sequence of the cell attachment sequence (RGD) is marked by asterisks. The multicopper oxidase signature (HCHLERHSSWGM) is boxed. Three copper oxidase‐like domains are underlined. Dots represent gaps introduced to maximize similarities.

**Fig. S3** Expression of *GhLAC15 *in different tissues (root, stem and leaf), tested through quantitative reverse transcription‐polymerase chain reaction (qRT‐PCR). *GhActin* was used as an internal control. Data are presented as average values with standard deviation (*n* = three technical replicates).

**Fig. S4** Primer efficiencies for *GhLAC15* and *GhActin* primer pairs.

**Fig. S5** Correlation between *GhLAC15 *gene expression level and corresponding disease resistance in silenced seedlings.

**Table S1** Primers used in this study.

**Table S2** Cotton varieties used for *GhLAC15 *gene expression analysis.
