# Supplementary figures and images for: The cotton laccase gene GhLAC15 enhances Verticillium wilt resistance via an increase in defence‐induced lignification and lignin components in the cell walls of plants
Source: Mol Plant Pathol. 2018 Nov 15;20(3):309–22. doi: 10.1111/mpp.12755 (PMC6637971; doi:10.1111/mpp.12755)

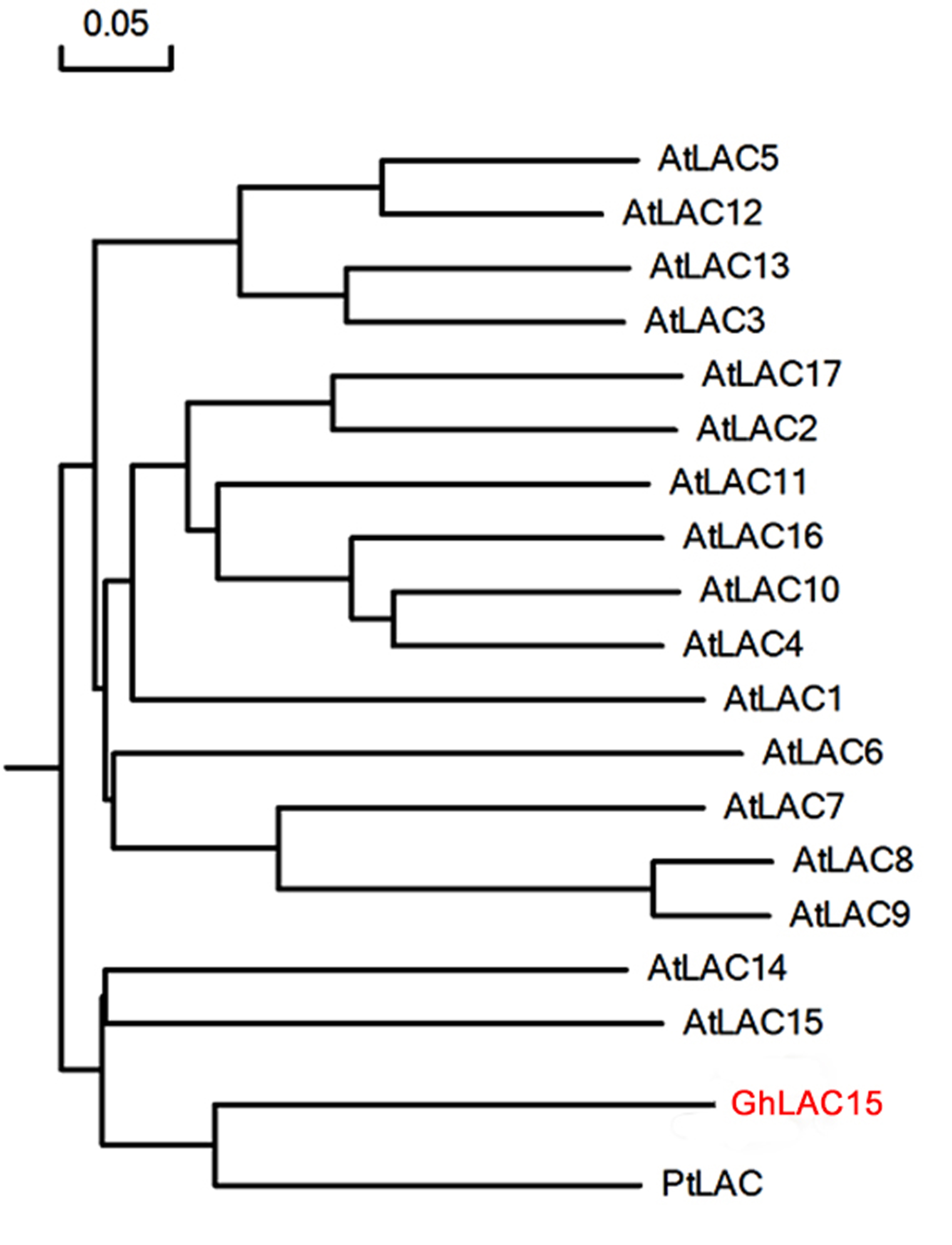

Supplement: Supplementary file 1 — Fig. S1 Phylogenetic analysis of laccase proteins. Phylogenetic relationship of GhLAC15, AtLAC proteins and two other plant laccases. The phylogenetic tree was constructed using DNAMAN6.0 Multiple Sequence Alignment programs. GhLAC15 is marked in red. The two letters preceding the protein name describe the organism from which the sequence was derived: At, Arabidopsis thaliana; Pt, Populus trichocarpa. LAC, laccase. AtLAC1 (NM_101674), AtLAC2 (NM_128470), AtLAC3 (NM_128574), AtLAC4 (NM_129364), AtLAC5 (NM_129597), AtLAC6 (NM_130222), AtLAC7 (NM_111756), AtLAC8 (NM_120181), AtLAC9 (NM_120182), AtLAC10 (NM_120197), AtLAC11 (NM_120404), AtLAC12 (NM_120621), AtLAC13 (NM_120795), AtLAC14 (NM_120972), AtLAC15 (NM_124184), AtLAC16 (NM_125281), AtLAC17 (NM_125395), PtLac (XM_002325536). The bar indicates the relative branch length. GhLAC15 and GhLAC17 laccases from Arabidopsis were used to build the tree. The branch length is proportional to the number of substitutions per site and represents evolutionary distance, as indicated by the scale bar. [file MPP-20-309-s001.jpg]

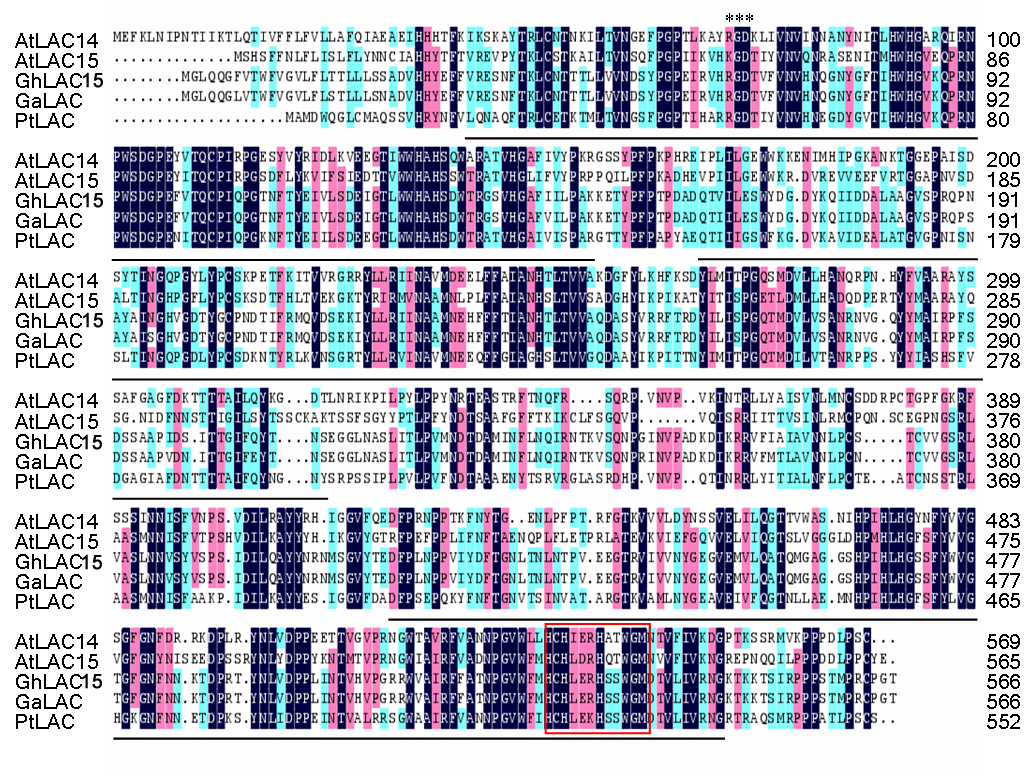

Supplement: Supplementary file 2 — Fig. S2 Multiple alignment of the deduced amino acid sequences of GhLAC15 and other plant laccases. The amino acid sequences of GhLAC15, GaLAC, PtLAC and Arabidopsis thaliana (AtLAC4 and AtLAC17) were aligned with ClustalW software. The consensus sequence of the cell attachment sequence (RGD) is marked by asterisks. The multicopper oxidase signature (HCHLERHSSWGM) is boxed. Three copper oxidase‐like domains are underlined. Dots represent gaps introduced to maximize similarities. [file MPP-20-309-s002.jpg]

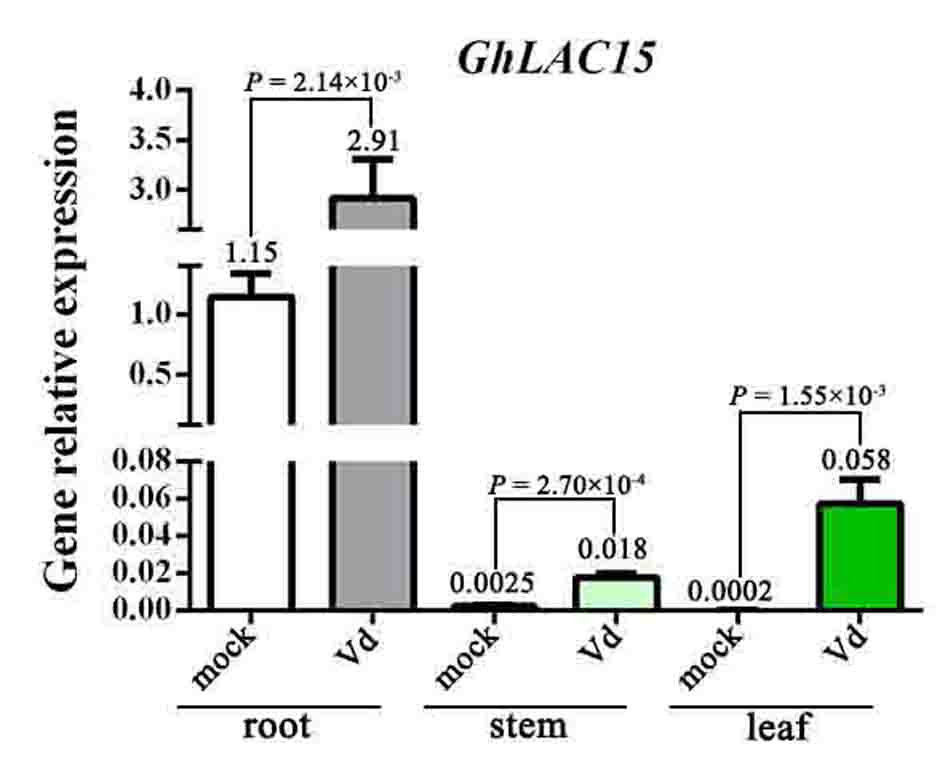

Supplement: Supplementary file 3 — Fig. S3 Expression of GhLAC15 in different tissues (root, stem and leaf), tested through quantitative reverse transcription‐polymerase chain reaction (qRT‐PCR). GhActin was used as an internal control. Data are presented as average values with standard deviation (n = three technical replicates). [file MPP-20-309-s003.jpg]

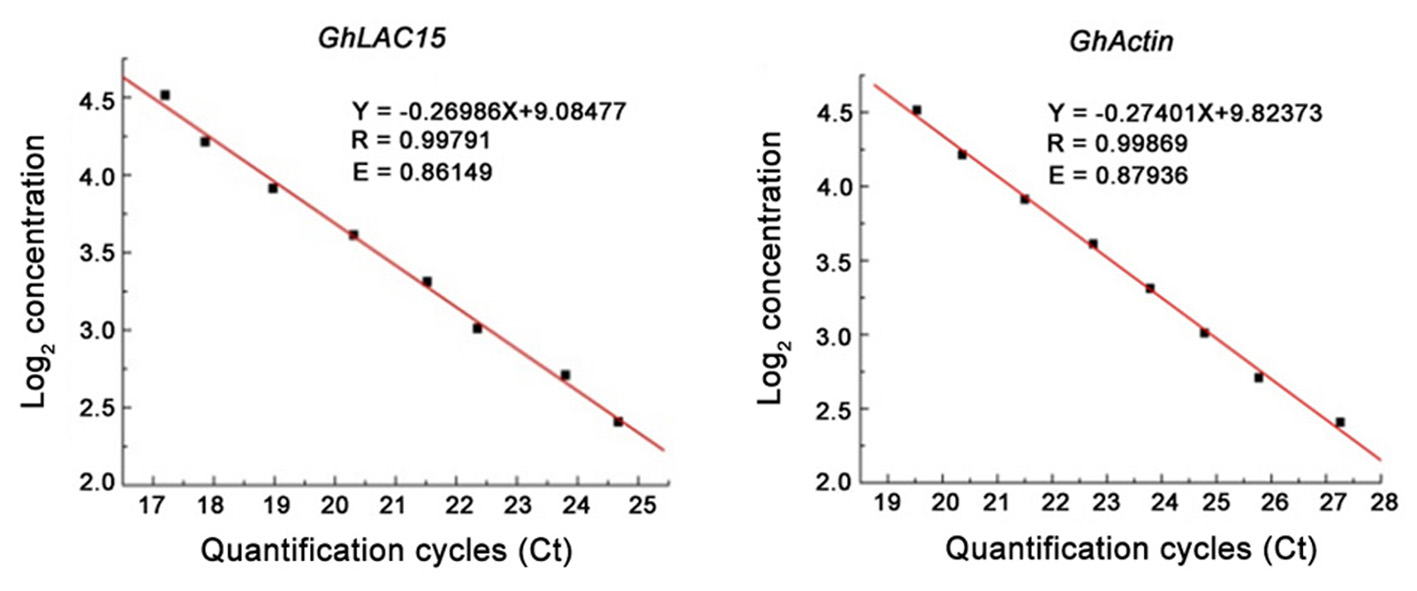

Supplement: Supplementary file 4 — Fig. S4 Primer efficiencies for GhLAC15 and GhActin primer pairs. [file MPP-20-309-s004.jpg]

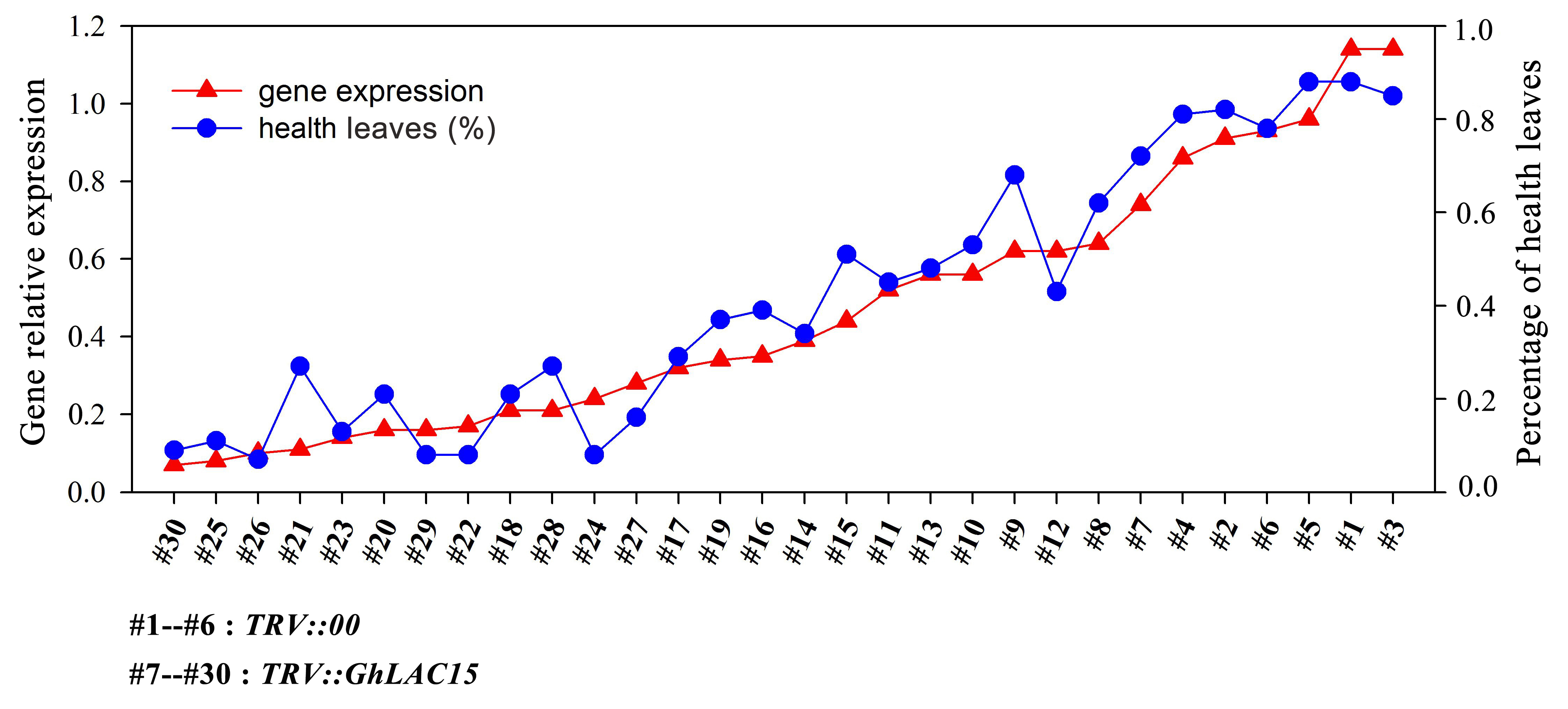

Supplement: Supplementary file 5 — Fig. S5 Correlation between GhLAC15 gene expression level and corresponding disease resistance in silenced seedlings. [file MPP-20-309-s005.jpg]
