# Supplementary material for: The cotton laccase gene GhLAC15 enhances Verticillium wilt resistance via an increase in defence‐induced lignification and lignin components in the cell walls of plants
Source: Mol Plant Pathol. 2018 Nov 15;20(3):309–22. doi: 10.1111/mpp.12755 (PMC6637971; doi:10.1111/mpp.12755)
Supplement: Supplementary file 6 — Table S1 Primers used in this study. [file MPP-20-309-s006.docx]

Table S1 Primers used in this study

| Primers name | Primers sequence | [Application](javascript:void(0);) |
| --- | --- | --- |
| LacF1 | 5'-GGGTACCATGGGTTTACAGCAAGGTTTTG-3' | ORF |
| LacR1 | 5'-CTCTAGACTAGGTTCCAGGACACCG-3' |  |
| LacrF2 | 5'-GGGAAATGGGTTTACAGCAAGG-3' | Real-time |
| LacrR2 | 5'-GACCAGCAATGTGGTTGTGTTG-3' |  |
| LacF3 | 5'-GTCGACATGGGTTTACAGCAAGGTTTTGTG-3' | pCamLac::GFP |
| LacR3 | 5'-GGTACCGGTTCCAGGACACCGAGGCATG-3' |  |
| LacF4 | 5'-GAATTCAATCCCGGAGTATGGTTTATGC-3' | pTRV-GhLAC |
| LacR4 | 5'-GGGTACCCTTTCATTGTTTTTGCTTGATTTGC-3' |  |
| GhActinF | 5'-ATACCGTCCTGGAACTGTTGCTCT-3' | Real-time |
| GhActinR | 5'-TTCAAAAAGACCCACAAGGTATGC-3' |  |
