# Supplementary material for: The cotton laccase gene GhLAC15 enhances Verticillium wilt resistance via an increase in defence‐induced lignification and lignin components in the cell walls of plants
Source: Mol Plant Pathol. 2018 Nov 15;20(3):309–22. doi: 10.1111/mpp.12755 (PMC6637971; doi:10.1111/mpp.12755)
Supplement: Supplementary file 7 — Table S2 Cotton varieties used for GhLAC15 gene expression analysis. [file MPP-20-309-s007.docx]

Table S2 Cotton varieties used for *GhLAC15* gene expression analysis.

| **Tolerant** | **Disease index** | **Susceptible** | **Disease index** |
| --- | --- | --- | --- |
| Tang mian7401 | 22.9 | Jin zhou tui hua mian | 54.6 |
| Zhong1421 | 24.0 | Ren dong67-86 | 61.9 |
| Su yuan04-3 | 29.0 | 73-782 | 62.6 |
| Jin mian20 | 28.6 | Nong lin1 | 67.5 |
| Yu mian21 | 26.2 | Xu zhou1818 | 67.9 |
| Su mian22 | 30.0 | Xin lu zao28 | 66.3 |
| CC28 | 26.4 | 73-184 | 76.6 |
| Ku che96515 | 26.8 | Jun mian1 | 70.5 |
